# Predictive Study on the Occurrence of Wheat Blossom Midges Based on Gene Expression Programming with Support Vector Machines

**DOI:** 10.3390/insects15070463

**Published:** 2024-06-21

**Authors:** Yin Li, Yang Lv, Jian Guo, Yubo Wang, Youjin Tian, Hua Gao, Jinrong He

**Affiliations:** 1 College of Information Engineering, Northwest A&F University, Yangling, Xianyang 712100, China; ly@nwsuaf.edu.cn; 2 Shaanxi Engineering Research Center of Agriculture Information Intelligent Perception and Analysis, Yangling, Xianyang 712100, China; 3 College of Mechanical and Electronic Engineering, Northwest A&F University, Yangling, Xianyang 712100, China; lvyang@nwafu.edu.cn (Y.L.); gjian@nwafu.edu.cn (J.G.); 4 College of Economics and Management, Northwest A&F University, Yangling, Xianyang 712100, China; wangyb03@nwafu.edu.cn (Y.W.); tyj@nwafu.edu.cn (Y.T.); 5 College of Horticulture, Northwest A&F University, Yangling, Xianyang 712100, China; 6 College of Mathematics and Computer Science, Yan’an University, Xi’an 716000, China

**Keywords:** smart agriculture, pest prediction, pest management, machine learning, gene expression programming–support vector machines, hierarchical classification tasks

## Abstract

**Simple Summary:**

In this study, we tackled an important issue in modern farming: predicting plant pests and diseases more effectively. Traditional methods are slow and often incorrect. To improve this, we created a new method that combines two advanced techniques, named gene expression programming (GEP) and support vector machines (SVM). Think of it as creating a smart program that can learn from past pest attacks to better predict future ones. We tested our new method with data on wheat pests from Shaanxi Province, recorded from 1933 to 2010. By comparing our method to other traditional ones, we found that ours was more accurate, with a success rate of about 91% in capturing pest attacks. This means our method can help farmers understand and prepare for pest and disease threats more efficiently, saving time and resources. Our work is a step forward in making farming smarter and more prepared for challenges, which is great news for ensuring our food supplies are secure and sustainable.

**Abstract:**

This study addresses the challenges in plant pest and disease prediction within the context of smart agriculture, highlighting the need for efficient data processing techniques. In response to the limitations of existing models, which are characterized by slow training speeds and a low prediction accuracy, we introduce an innovative prediction method that integrates gene expression programming (GEP) with support vector machines (SVM). Our approach, the gene expression programming—support vector machine (GEP-SVM) model, begins with encoding and fitness function determination, progressing through cycles of selection, crossover, mutation, and the application of a convergence criterion. This method uniquely employs individual gene values as parameters for SVM, optimizing them through a grid search technique to refine genetic parameters. We tested this model using historical data on wheat blossom midges in Shaanxi Province, spanning from 1933 to 2010, and compared its performance against traditional methods, such as GEP, SVM, naive Bayes, K-nearest neighbor, and BP neural networks. Our findings reveal that the GEP-SVM model achieves a leading back-generation accuracy rate of 90.83%, demonstrating superior generalization and fitting capabilities. These results not only enhance the computational efficiency of pest and disease prediction in agriculture but also provide a scientific foundation for future predictive endeavors, contributing significantly to the optimization of agricultural production strategies.

## 1. Introduction

The accurate prediction and timely management of plant pests and diseases are crucial for ensuring sustainable agriculture, food security, and environmental protection. The advent of advanced technologies, especially in the field of data analytics and machine learning, has provided new opportunities for enhancing the prediction and management of these agricultural threats. However, several challenges persist, such as the complexity and heterogeneity of data related to plant health, the dynamic and evolving nature of pests and diseases, and the limited generalizability of existing prediction models. A primary challenge is the complexity and heterogeneity of data related to plant health. This includes diverse data sources, such as satellite imagery, weather data, soil conditions, and historical pest and disease records. Integrating and analyzing these multifaceted data to generate accurate predictions remains a daunting task. Plant pests and diseases are dynamic and continuously evolving, adapting to changes in climate, agricultural practices, and host plant resistance [1]. The dynamic nature of pests and diseases makes it challenging to develop models that can accurately predict future outbreaks and their severity. Many existing prediction models are developed for specific regions or crops and may not be easily generalizable to other contexts. This limitation affects their applicability and scalability, especially in regions with diverse agricultural practices and environmental conditions. Timely and accurate predictions are essential for effective pest and disease management. However, developing real-time prediction models and early warning systems that can provide actionable insights to farmers and agricultural stakeholders remains a challenge.

Recent years have witnessed significant advancements in predictive and classification models for plant pest and disease prediction. Machine learning techniques, including random forests, support vector machines (SVM), and neural networks, are increasingly being applied to develop models that can handle complex data and provide accurate predictions. For example, Patra et al. (2023) utilized a Random Forest algorithm to predict the occurrence of plant diseases with high accuracy [2]. This aligns with the findings from another study that employed Random Forest to distinguish between healthy and diseased leaves from datasets created specifically for this purpose [3]. The effectiveness of Random Forest in plant disease prediction is further supported by its application in various studies, highlighting its robustness and efficiency in classifying different types of diseases [4,5].

SVM is renowned for its robustness and effectiveness in classification tasks, particularly in high-dimensional spaces, as evidenced by its successful application in classifying sugar beet diseases using hyperspectral imaging [6]. One study demonstrated the use of an improved deep learning feature extraction algorithm combined with a particle swarm SVM model for crop disease prediction, achieving high precision rates of up to 0.84 [7]. Combining image processing techniques with SVM has also proven effective in predicting pests and diseases. By leveraging mathematical morphology features extracted from images, researchers have developed dynamic prediction models that achieve high prediction accuracies, such as 90% [8]. This approach addresses the limitations of traditional methods by providing more accurate and scientific predictions. SVM’s ability to distinguish between diseased and healthy leaves with high fitting and predictive precision has been well-documented, making it a valuable tool in crop disease diagnosis [9]. Furthermore, SVM has been extensively used for disease classification, demonstrating its versatility and effectiveness in different agricultural contexts [10].

Neural networks, especially convolutional neural networks (CNNs), have also been explored for their potential in predicting plant diseases. Fang et al. (2018) showcased the ability of a convolutional neural network to successfully identify apple leaf diseases [11]. The development of deep learning models, such as enhanced CNNs combined with long short-term memory (LSTM) networks, has shown promising results in detecting plant pests and diseases with high accuracy [12]. These models leverage deep feature extractions and ensemble classifiers to improve detection and classification performance.

The integration of different machine learning techniques has led to the development of hybrid models that offer enhanced performance in terms of accuracy and speed. For example, an improved RBF neural network combined with fuzzy clustering was proposed to enhance the prediction of pests in small sample sizes, showing better generalization capabilities and increased practicality [13]. Similarly, models that combine rough set theory with artificial neural networks have been developed to improve training time and prediction accuracy [14].

Transfer Learning and Model Optimization: Transfer learning has been utilized to enhance the robustness and classification accuracy of pest and disease detection models. By pre-training models on large datasets and fine-tuning them on specific agricultural pest datasets, researchers have achieved higher classification accuracies [15,16]. Additionally, model optimization techniques such as knowledge distillation and neural network quantization have been applied to accelerate model inference speeds while retaining high accuracy [17].

Among the various approaches, genetic expression programming (GEP) has become a powerful tool in the realm of plant pest and disease prediction. GEP combines the advantages of genetic algorithms and genetic programming, enabling the development of models that can evolve and adapt over time. This feature is particularly beneficial in plant pest and disease prediction, where the ever-changing threats require models that can continuously update and improve. GEP has proven its effectiveness in various studies related to plant health prediction, showcasing its ability to handle complex agricultural data and develop models that are generalizable across different crops and regions. GEP’s rapid model evolution and updating capabilities make it a promising approach for developing real-time prediction systems and early warning mechanisms [18,19,20,21].

In this study, we aim to address the challenges associated with plant pest and disease prediction by proposing a novel approach that combines GEP with SVM. By integrating GEP and SVM, our proposed model, the GEP-SVM model, leverages the strengths of both approaches: GEP’s adaptability and evolutionary dynamics, alongside SVM’s exceptional classification accuracy. This combination is particularly useful for creating a robust model that is capable of adapting to new threats while maintaining high precision in in hierarchical classification tasks, thus providing a comprehensive solution to the challenges faced in plant pest and disease prediction.

## 2. Construction of GEP-SVM-Based Model for Plant Pest Prediction

### 2.1. Theory of GEP Algorithm

GEP is a genetic algorithm variant that utilizes a tree structure to represent expressions. Unlike genetic programming, which uses a genetic coding represented through expressions, GEP uses a series of symbol strings in its genetic code. The key feature of GEP is its use of K-expressions, linear strings that represent predictive functions, allowing for the inter-transformation between expression trees and functional expressions. This ability is particularly relevant to our study as it enhances the flexibility and efficiency of prediction algorithms. The focus of this text will be on algorithms for the inter-transformation of expression trees, functional expressions, and K-expressions, highlighting their role in gene expression programming for prediction algorithms.

#### 2.1.1. GEP Algorithm

**Figure 1:**
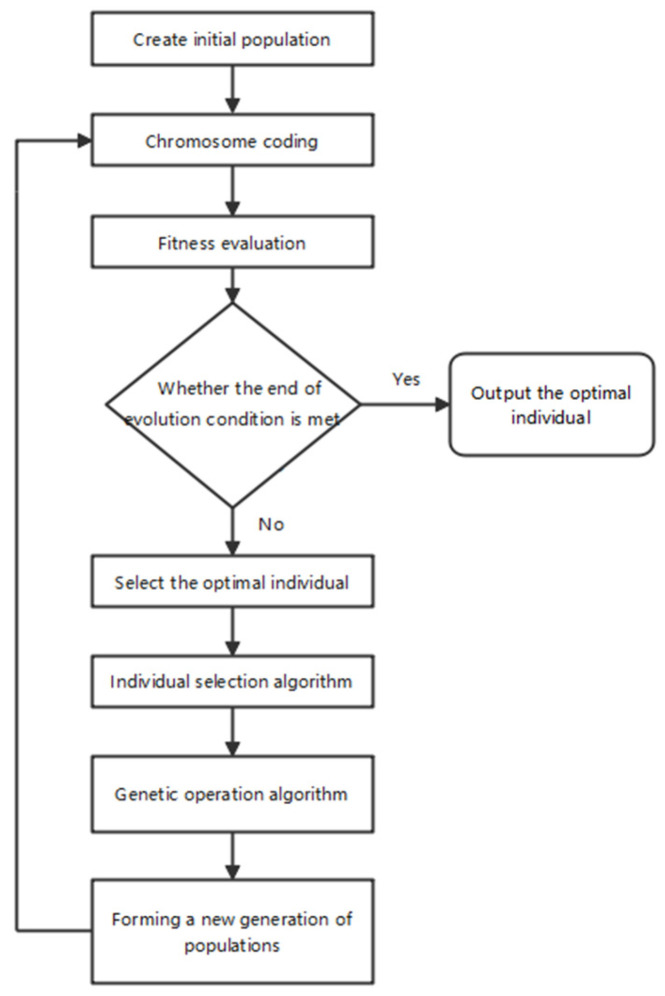
Process flow diagram of the GEP algorithm.

In our study, we utilize the gene expression programming algorithm, which is guided by the principle of ‘the survival of the fittest’ in biological evolution. The algorithm evaluates the fitness of individuals in a population and iteratively selects and genetically modifies superior individuals until an optimal solution that meets the termination criteria is found (Figure 1).

#### 2.1.2. Fitness Functions

In gene expression programming, the following fitness function is commonly used to assess the fitness of an individual:

(1) $$f_{i}=\sum_{j=1}^{C_{t}}\left(\frac{\left|M-C_{\left(i,j\right)}-T_{j}\right|}{T_{j}}\right)$$

(2) $$f_{i}=\sum_{j=1}^{C_{t}}\left(\left|M-C_{\left(i,j\right)}-T_{j}\right|\right)$$

Equation (1) is used when dealing with relative error problems and Equation (2) is used when dealing with absolute error problems. The letters in the equations are described in Table 1.

#### 2.1.3. Genetic Operators

Genetic modification can induce changes in the genetic characteristics of a population and enhance its diversity. This can facilitate the evolution of individuals within populations by introducing genetic operators. Specific and distinctive genetic operators can be designed for various practical problems.

(1) Selection and Replication

During natural evolution and biological inheritance, species with a higher adaptability to their environment have a greater probability of their traits being inherited by the next generation, while those with lower adaptability have a relatively lower probability. Imitating this process, GEP applies selection or replication operators to “winnow” individuals within a population.

(2) Mutation

Following the example of natural evolution, gene expression programming introduces mutation operators to prompt the emergence of new individuals. Mutations can occur in any part of the chromosome. During mutation, the head element can mutate into a function or a terminator, while the tail element can only mutate into a terminator.

(3) Transfer

In gene expression programming, three types of transfer operators are generated based on different transfer factors, as shown in Table 2.

In gene transfer, the selected gene becomes the transfer factor and is transferred to the beginning of the chromosome, becoming the first gene. Overall, the transfer operator is less effective than the mutation operator, but it is still frequently used in GEP.

(4) Recombination

Recombination genetic operators are divided into genetic recombination, single-point recombination, and two-point recombination. They share a common principle: two paired chromosomes are randomly selected, recombination points are randomly determined at identical positions, and then the sequences of the two parent chromosomes are exchanged. In genetic recombination, the two parent chromosomes swap genes at identical locations. The recombination operator is slightly less effective than the transfer operator, and both are less effective compared to the mutation operator. In single-point recombination, a random recombination point is identified on the paired chromosomes, and all sequences beyond this point are exchanged. In contrast, in two-point recombination, there are two recombination points, and the sequences between these points are exchanged.

### 2.2. GEP-SVM Algorithm

#### 2.2.1. Gene Expression Programming–Support Vector Machine Algorithm

**Figure 2:**
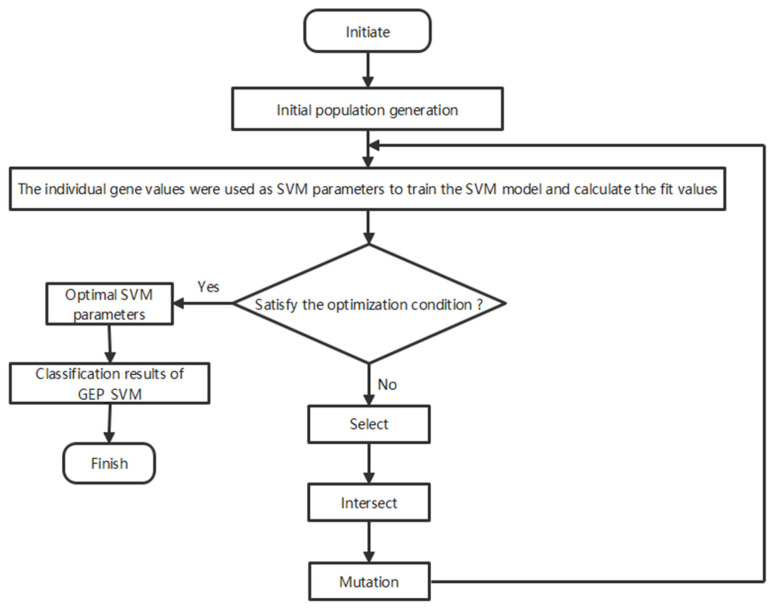
Operational flowchart of the GEP-SVM hybrid algorithm for disease classification.

The operation of the GEP-SVM algorithm consists of the following: performing encoding, determining the fitness function, forming the initial solution, entering the computational model through operations such as looping, selection, intersection, mutation, and convergence criteria. The genetic parameters with optimal characteristics are obtained by rotating the orthogonal way, the individual gene values are used as SVM parameters, the SVM model is trained, the fitness is determined, and finally the gene expression programming–support vector machine disease prediction algorithm is obtained. The flow of the prediction algorithm is shown in Figure 2.

(1) Encoding

When using the binary coding method for encoding, the length of the symbol string is first determined according to the solution accuracy required by the problem, assuming that the range of values of a certain parameter is [$X_{\min}$, $X_{\max}$] and the parameter can be represented by the binary coding symbols of length L, which can produce 2L different codes. Let the coding accuracy be δ; then:

$X_{\min}$ is denoted as 0000.... 000 represents 0

$X_{\max}$ is denoted as 1111.... 111 represents 2L − 1

(3) $$\frac{X_{\max}-X_{\min}}{2^{L}-1}$$

If an individual is encoded as X: a_L_a_L−1_a_L−2_…a_2_a_1_, then its decoding formula is:

(4) $$X\min+\left(\sum_{i=1}^{L}a_{i}\cdot 2^{i-1}\right)\cdot \frac{X\max-X_{\min}}{2^{L}-1}$$

(2) Determine the fitness function

Because the programming of gene expression can only be found in the great value, and reactive optimization is a problem of finding the very small value, the objective function needs to be transformed to change the seeking of the very small value into the seeking of the great value. This can be achieved through the following equation:

(5) $$\max\left(x-f\right)$$

The fitness function is:

(6) $$fit=1000-\left(\sum P_{L}\left(x\right)+\lambda_{1}\sum {\left(\frac{V_{i}-V_{ilim}}{V_{imax}-V_{imin}}\right)}^{2}+\lambda_{2}\sum {\left(\frac{Q_{i}-Q_{ilim}}{Q_{imax}-Q_{imin}}\right)}^{2}\right)$$

(3) Formation of the initial solution

An array of individuals of number n, with each individual being one, can be randomly generated as follows:

(7) $$\left[V_{1},V_{2},\cdots V_{n1},T_{1},T_{2},\cdots T_{n2},Q_{c1},Q_{c2}\cdots Q_{cn3}\right]$$

(4) The loop used to calculate the tidal current is as follows:

The trend is calculated for each individual, and the result is substituted into the fitness function to obtain the corresponding fitness function value. By looping n times, n fitness values are obtained, from which the largest fitness function value is selected $fit_{max}^{\left(k\right)}$.

(5) Selection

The obtained n fitness values are sorted from largest to smallest, the last 1/4 is replaced with the top 1/4, and n individuals are re-formed. In this way, the better individuals are selected, and the diversity of the population is ensured.

(6) Crossover

The crossover loop with the crossover rate $P_{c}$ set to 0.9 is entered. Generate a random number between 0 and 1. If the number is less than $P_{c}$, perform the crossover; otherwise, keep it the same. Loop n/2 times without repeating the crossover. The following is the crossover formula.

The real variable crossover formula is as follows:

(8) $$\left\{\begin{array}{l} X\prime_{i}=\left(1-a\right){\times X}_{i}+a\times X_{j}\\ X\prime_{j}=a{\times X}_{i}+\left(1-a\right){\times X}_{j} \end{array}\right.$$

The integer variable crossover formula is as follows:

(9) $$\left\{\begin{array}{l} X\prime_{i}=round\left[\left(1-a\right)X_{i}+aX_{j}\right]\\ X\prime_{j}=round\left[aX_{i}+\left(1-a\right)X_{j}\right] \end{array}\right.$$

Note that $X_{i}$, $X_{j}$ represent the two bodies to be crossed; $X\prime_{i}$, $X\prime_{j}$ represent the two newly generated bodies; a is a randomly generated number between 0 and 1.

(7) Mutation

The mutation loop is entered. Here, the mutation rate $P_{m}$ is set to 0.1. A number between 0 and 1 is randomly generated. If this number is less than $P_{m}$, it is mutated; otherwise, it remains unchanged. At the time of mutation, a random binary code string (masked word) is generated. The cycle is repeated n times and n new individuals are formed.

The mutation formula is:

(10) $${X^{\prime }}_{i}=\left\{\begin{array}{c} X_{i}-\left(X_{i}-X_{imin}\right)\times b\;\;Position\;corresponding\;to\;0\\ X_{i}-\left(X_{imax}-X_{i}\right)\times b\;\;Position\;corresponding\;to\;1 \end{array}\right.$$

$X_{i}$ is the individual to be mutated; ${X^{\prime }}_{i}$ is the new individual produced by the mutation; b is a randomly generated number between 0 and 1.

(8) Convergence criterion

For the latest formation of n individuals, the trend calculation is performed, and the result is substituted into the fitness function to obtain n fitness function values, from which the largest fitness function value $fit_{max}^{\left(k+1\right)}$ is selected. If $fit_{max}^{\left(k+1\right)}-fit_{max}^{\left(k\right)}<\varepsilon$ (ε is a very small number set in advance), the convergence criterion is satisfied and the result is output. Otherwise, the steps are repeated from step 4 until the convergence criterion is satisfied or the maximum number of iteration generations is reached. The algorithm of GEP-SVM is used to set the fitness function and implements several evolutionary operator operations such as selection, crossover, mutation, string insertion, recombination, extraction, etc., to the population, so that the population evolves from generation to generation. The algorithm also searches for the optimal individuals, thus obtaining a better prediction model. The pseudo-code is shown in Table 3.

#### 2.2.2. Integration and Data Processing in GEP-SVM

**Figure 3:**
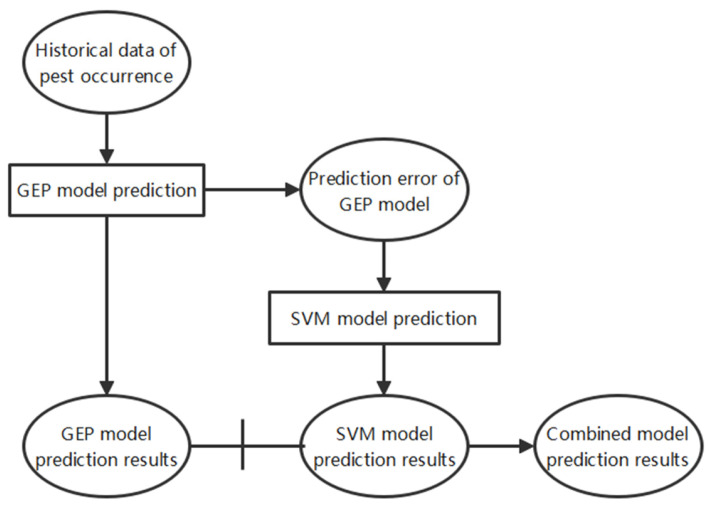
Process flow diagram of the GEP-SVM hybrid forecasting model for pest occurrence prediction.

When using a single model (SVM or GEP) for prediction in pest prediction work, the final results are likely to reflect the problem of excessive error. Therefore, this study proposes the use of a combined GEP-SVM model for pest prediction, and its prediction process is shown in Figure 3. As depicted in Figure 3 and Table 3, our model initially utilizes the historical data of pest occurrence to build the GEP prediction model. The prediction results of the GEP model capture the linear relationships present in the historical data. Additionally, the residuals from the GEP model predictions, which represent the nonlinear patterns not captured by the GEP model, are subsequently processed by the SVM model. The SVM model is specifically chosen for its ability to model these nonlinear patterns effectively. The prediction results of the SVM model thus encapsulate these nonlinear characteristics. Finally, the outputs from both models are combined to produce the final predicted values. This fusion leverages the strengths of both models: the GEP model’s proficiency in capturing linear relationships and the SVM model’s ability to handle nonlinear patterns, resulting in a comprehensive and accurate prediction model.

#### 2.2.3. Model Evaluation Metrics

The predictive ability of a model denotes its capacity to accurately represent samples not included in the training set (i.e., the test sample set). The predictive ability of the model is crucial for determining its practical applicability; a strong predictive ability denotes greater practical value. At present, various metrics are employed to assess the predictive ability of models. In this study, we primarily utilize the Mean Square Error (MSE) and Mean Absolute Percentage Error (MAPE) as our evaluation metrics. These metrics were selected because MSE provides a measure of the average squared difference between the predicted and actual values, offering a clear indication of the model’s accuracy. On the other hand, MAPE expresses the prediction error as a percentage, providing an intuitive understanding of the model’s performance relative to the scale of the data. The combination of these two metrics allows for a comprehensive evaluation of the GEP-SVM model’s performance in plant pest and disease prediction, as demonstrated in Table 4.

The evaluation indices of the model mainly include the MSE and MAPE indices, as mentioned above. In addition, we also use accuracy to evaluate the prediction results. We use 0.5 as the level threshold, with decimal parts greater than 0.5 corresponding to the next level and those less than 0.5 corresponding to the current level. The usual formula for the accuracy rate is $AC=\frac{M}{N}$, where M represents the number of samples whose predicted grade is equal to the actual grade and N represents the total number of samples. Considering the potential errors in field surveys of wheat blossom midges, this paper proposes the use of Formula (11):

(11) $$AC=\frac{M+D\times 0.5}{N}$$

where M represents the number of samples in which the predicted grade is equal to the actual grade, D represents the number of samples in which the predicted grade differs by one level from the actual grade, and N represents the total number of samples.

In addition, we used Precision, Recall, F1−Score, and the Matthews Correlation Coefficient (MCC) as evaluation indicators. The formulas for these metrics are as follows:

(1) Precision: This measures the accuracy of positive predictions. It is defined as the ratio of true positive predictions to the total number of positive predictions made. The formula is:

(12) $$\mathrm{Precision}=\frac{TP}{TP+FP}$$

(2) Recall (also known as Sensitivity or True Positive Rate): This measures the ability of the model to identify all relevant instances. The formula is:

(13) $$\mathrm{Recall}=\frac{TP}{TP+FN}$$

(3) F1−Score**:** This is the harmonic mean of Precision and “Recall” and is used as a balance between them. It is particularly useful when the classes are imbalanced. The formula is:

(14) $$F1-Score=2\times \frac{Precision\times Recall}{Precision+Recall}$$

(4) MCC: This is a more informative measure than F1−Score when evaluating binary classifications, as it takes into account true and false positives and negatives. It is generally regarded as a balanced measure, which can be used even if the classes are of very different sizes. The formula is:

(15) $$MCC=\frac{\left(TP\times TN\right)-\left(FP\times FN\right)}{\sqrt{\left(TP+FP\right)\left(TP+FN\right)\left(TN+FP\right)\left(TN+FN\right)}}$$

where TP denotes true positives, FP denotes false positives, FN denotes false negatives and TN denotes true negatives.

## 3. Experimental Results and Analyses

Prediction and classification belong to the same search problem; prediction is for continuous attributes, while classification is for discrete attributes. GEP is most commonly used for prediction, grain yield prediction, and fault prediction, because GEP only evolves according to the fitness function, and accurate results can be obtained without human intervention. The long-term prediction of the extent of wheat sucker occurrence has a relatively large historical span. In this section, to ensure the efficiency and accuracy of the algorithm, we will establish a prediction model for the extent of wheat sucker occurrence based on GEP-SVM.

### 3.1. Data Preparation and Data Description

The meteorological data spanning 1933 to 2010 used in this study were obtained from the meteorological station in Xi’an City, Shaanxi Province. The data on the occurrence of wheat suckers were sourced from the Institute of Plant Protection of Shaanxi Academy of Agricultural Sciences, as detailed in Table 4. Meteorological factors, particularly rainfall and temperature, are another major cause of disaster occurrence. Through a correlation analysis of rainfall, temperature, and the population of aphids from 1952 to 1992, Li Xiulian and others [22] demonstrated that the occurrence of wheat aphids in Shaanxi’s Guanzhong region is negatively correlated with the temperatures in January, February, and March. Conversely, it shows a significant positive correlation with the rainfall in the July, August, and September of the previous year, as well as the January of the current year. The rainfall trends in July and August from 1954 to 1992 reveal that during the 1950s and early 1980s, when aphid outbreaks were more severe, there was notably higher precipitation; in contrast, 1986 and 1987, which witnessed the lowest occurrence of aphids, had the least rainfall in the July and August of the preceding year among all recent years.

The grading of the occurrence degree of wheat suckers followed the method proposed by Professor Yuan Feng. The grading rules are as follows: Level 1 indicates that there were no infested fields, and no reports of infestation were recorded. Level 2 indicates that 1–10 infested fields were observed in 1–2 counties or more, or there were historical records or reports of infestation in 1–2 districts and counties. Level 3 indicates that there were 10 or more infested fields in each of more than 3 counties, or there were records or reports of infested fields in more than 3 districts and counties.

The occurrence of wheat suckers is influenced by various factors, including meteorological conditions, the planting area of pest-resistant varieties, farming systems, irrigation conditions, and the base of the insect source. Among these, meteorological factors are among the most critical. Due to incomplete historical data on the base of the wheat sucker source and the inability to quantitatively analyze the planting area of early wheat varieties, farming systems, and irrigation conditions, these factors are not effectively utilizable for prediction. Consequently, in this study, only rainfall and temperature were employed as screening factors for predicting the incidence of wheat suckers, as shown in Table 5. It is important to note that our model is regression-based, focusing on predicting Incident Level or Occurrence Level based on these selected factors. As illustrated in Table 6, our decision to use a regression model stems from the inherently ordinal nature of our grading system. While the grades are discrete, they possess an inherent order (1 < 2 < 3) that reflects increasing levels of severity. In such cases, ordinal regression or ordered logistic regression models are often more appropriate than standard classification models, as they can leverage the ordinal relationship between the categories [23,24,25,26].

We classify Incident Level or Occupancy Level as Level 1, Level 2, Level 3, and so on. At this point, using only classification loss is not enough. If the true level of a sample is Level 1, using classification methods, the loss incurred when its level is classified into Levels 2 and 3 is equal. However, it is evident that Level 2 is closer to Level 1 than Level 3, making Level 2 a more acceptable classification than Level 3. Therefore, in terms of application, Level 2 should have a smaller loss than Level 3.

### 3.2. Experimental Environment

The algorithms in this experiment were implemented in the MATLAB language and were all executed on a PC with a CPU of Intel(R) Core (TM) i5-2450M CPU@2.50Hz, 4 GB of RAM using the operating system of Win7 Flagship (64-bit).

### 3.3. Data Pre-Processing

There is a close link between the occurrence of pests and natural factors, affected by both temperature and rainfall; the data collected in this paper are mainly composed of temperature and rainfall data. Normalization is a commonly used data pre-processing method. The data normalization process aims to remove the magnitude of the data to avoid the calculation process being increased due to the different magnitude of the larger error, as well as to increase the convergence speed of the model. The most commonly used normalization range is [23,24,25,26]; this experiment used the map minmax method in MATLAB to normalize the data to between [0,1]. This paper collected a total of 78 years of historical data; the 1933–1992 data were used as a training set; 1993–2010 data were used as a test set. Each year within our dataset is treated as an independent unit. There was no significant correlation between the years, ensuring that the test set provided an unbiased evaluation of our model’s performance.

### 3.4. Parameter Setting

The operating parameters of the GEP-SVM model are shown in Table 7.

### 3.5. Experimental Analysis

The results of the prediction of wheat blossom midges’ occurrence class based on the six types of models established by plain Bayes, BP neural network, K nearest neighbors, SVM, standard GEP, and GEP-SVM were compared and analyzed in two ways: a performance analysis of GEP-SVM and a comparative analysis of the prediction data.

#### 3.5.1. Optimization of GEP-SVM Parameters

Based on the 5-fold cross-validation method, the multi-class SVM classifier was trained to find the optimal combination of classifiers c and g. The number of iterations was set to 100 at the very beginning, and then, according to the adaptation curve that was obtained, the minimum number of iterations that tends to be stable under the condition of optimal appropriateness was used as the final parameter setting for the GEP algorithm. The number of populations was 30.

**Figure 4:**
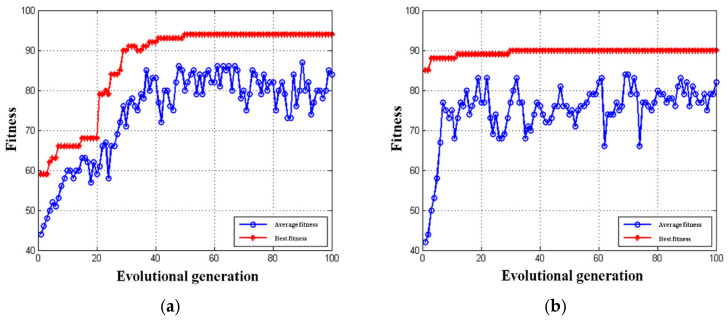
Tracking the optimization convergence of the GEP-SVM algorithm across 100 generations: (**a**) iterations of a small number of populations; (**b**) iterations of a large number of populations.

From Figure 4a, it can be seen that, for a small number of populations, when the number of iterations was 30, the best fitness of the GEP parameter seeking optimization already tended to be stable and the optimization could be stopped; therefore, in order to reduce the required time, the number of iterations in this paper was set to 30.

Similarly, from Figure 4b, it can be seen that for a large number of populations, when the number of iterations is 50, the best fitness of the GEP parameter seeking optimization already tended to be stable and the optimizations can be stopped; therefore, for this group of features, the number of iterations was selected to be 50. Stabilized and optimizations can be stopped; therefore, for this group of features, the number of iterations in this paper was chosen to be 50.

#### 3.5.2. Model Performance Analysis

To rigorously evaluate the predictive performance of the GEP-SVM model concerning wheat sucker incidence, this study conducted a series of comparative experiments against several well-established predictive models. These models included GEP, SVM, Naive Bayes, K-Nearest Neighbors (KNN), and the Back Propagation Neural Network (BPNN). Each model was selected based on its relevance and proven efficacy in similar domains of agricultural pest prediction. Specifically, the Naive Bayes algorithm was included due to its probabilistic approach to handling data uncertainty, KNN for its efficacy in capturing locality in data points, and BPNN for its robustness in learning complex patterns through its layered structure. Detailed descriptions of these algorithms are as follows:

(1) Naive Bayes: A probabilistic model that assumes feature independence and calculates the posterior probability of an outcome based on Bayes’ Theorem. It is especially effective when the dataset features are independent of each other, a condition approximated in our study due to the diverse and non-overlapping spectral data from wheat fields.

(2) KNN: A non-parametric algorithm that classifies a data point based on the majority class among its K closest neighbors. We chose KNN due to its simplicity and effectiveness in scenarios where the decision boundary is highly irregular.

(3) BPNN: A type of artificial neural network where the error between the actual and predicted outcomes is propagated backwards through the system to adjust model weights, thus optimizing performance during training. BPNN was selected for its capability to model the complex nonlinear relationships intrinsic to ecological and biological datasets.

These comparative assessments are intended not only to validate the robustness of the GEP-SVM model but also to highlight its superiority or potential limitations in forecasting the incidence of wheat sucker relative to other models.

Specifically, the GEP-SVM model employed a Radial Basis Function (RBF) kernel with a regularization parameter (C) of 10 and gamma set to 0.001. The GEP model parameters comprised a population size of 100, mutation rate of 0.02, and crossover rate of 0.8. For the SVM, a polynomial kernel with degree 3 and C set to 1 was utilized. The K-nearest neighbors model employed five neighbors, using a distance weight function and an auto-selection algorithm. The Naïve Bayes model did not adjust prior probability and utilized a Laplace smoothing of 1. Lastly, the BP Neural Network featured two hidden layers, containing 64 and 32 neurons, respectively, and employed a learning rate of 0.01 and a momentum of 0.9 for tuning.

The parameters of these six models underwent optimization through grid search techniques for hyperparameter tuning, and the models’ predictive capabilities underwent evaluation using MSE and MAPE, with the results being presented in Table 8. Table 8 presents the prediction error measures for wheat-sucker-affected fields, while Table 9 details the prediction error measures related to the occurrence degree, thus facilitating a comparison of the models’ accuracies.

As can be seen from the table of prediction errors for wheat suckers (Table 8), the fitting ability of GEP-SVM and GEP for the training set is significantly better than that of SVM, K-nearest neighbors, BP neural network, and plain Bayes. However, for the prediction of the test set, the prediction errors of GEP-SVM and GEP are significantly smaller than those of the remaining four models, indicating that using GEP to learn the features can effectively reduce the prediction errors. From the comparison of the back-generation test on the training set and the prediction of the prediction set, it can be seen that the BP neural network has the worst generalization ability.

**Figure 5:**
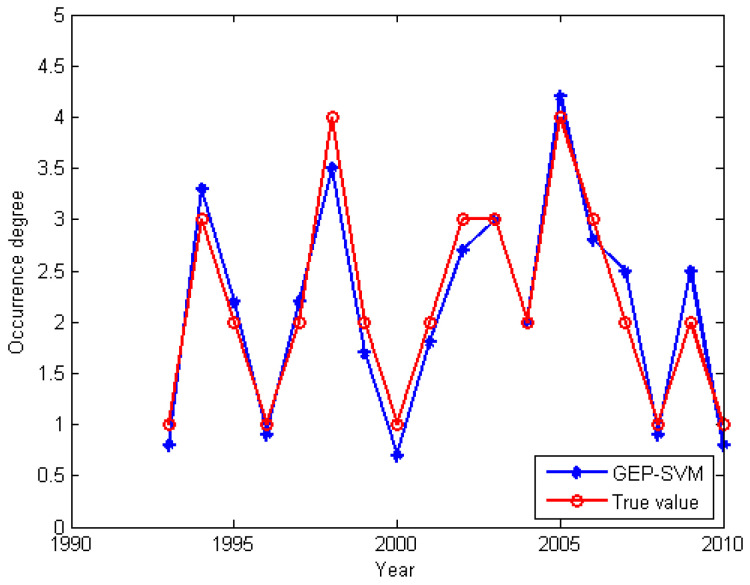
GEP-SVM prediction compared with real value.

In this study, we explored the use of various algorithms for plant pest and disease data processing. The detailed results presented in Table 9 and illustrated in Figure 5 highlight the performance of various predictive models for estimating the occurrence of wheat blossom midges. The GEP-SVM model demonstrates superior performance in both the training and testing phases. During the training phase, the GEP-SVM model achieved the highest accuracy of 90.83%, with a precision of 0.870, recall of 0.889, and F1-score of 0.880. It also scored an MCC of 0.501, indicating a strong positive relationship between the observed and predicted classifications. This model outperforms the standalone GEP and SVM models, which recorded accuracies of 88.33% and 87.50%, respectively, and slightly lower performance metrics across precision, recall, F1-score, and MCC.

In the testing phase, the GEP-SVM model again showcased its robustness, with an accuracy of 88.89%, the highest among the tested models. It exhibited a precision of 0.857, recall of 0.923, and an F1-score of 0.888, alongside an MCC of 0.563, underscoring its superior generalization ability when compared to other models. The standalone GEP and SVM models demonstrated accuracies of 80.55% each, with the GEP model having slightly better recall but lower precision than the SVM model.

The BP neural network, while exhibiting a larger error in the back-generation test for the training set and a lower accuracy regarding the occurrence degree, maintains a relatively small gap between its fitting ability for the training set and its prediction ability for the test set, indicating a better generalization ability. This is attributed to the employment of the kernel function and the minimization of structural risk.

On the other hand, the plain Bayesian model surpasses the BP neural network model in terms of predictive effectiveness. This can be attributed to the plain Bayesian model’s ability to effectively utilize the local information of the samples. However, when comparing the two models, GEP-SVM and GEP, there is not a significant difference in their effectiveness in performing back-generation tests on the training set. This similarity in performance is primarily due to both models employing GEP for automatic feature extraction, which results in the learning of new features that are superior to the original features and more relevant to the predictors.

Among the evaluated models, GEP-SVM demonstrates the best prediction ability. This superiority can be attributed to the combination of GEP’s strength in terms of feature extraction and SVM’s effectiveness in regression, which together enhance the prediction accuracy. This justifies our choice of the GEP-SVM model for this study, as it leverages the advantages of both GEP and SVM to provide an improved predictive performance in plant pest and disease prediction.

## 4. Performance Analysis of GEP-SVM Algorithm on Other Datasets

To better validate the performance of the GEP-SVM prediction algorithm, particularly its ability to handle the time-series data and enable the development of models that can evolve and adapt over time that were proposed in this study, two public datasets from China were selected: the manufacturing shipment dataset [27] and the traffic information dataset of a city section [28]. The performance of the GEP-SVM algorithm was evaluated and compared with other prediction algorithms, such as KNN, SVM, and GEP, utilizing model evaluation metrics.

### 4.1. Experimental Environment

The algorithms in this experiment were implemented in Python and executed on a PC with an Intel(R) Core (TM) i5-2450M CPU @ 2.50GHz, 4GB of RAM, and a Win7 Ultimate (64-bit) operating system.

### 4.2. Datasets

The experiments in this section used two main datasets: the manufacturing shipments dataset [27], sampled from February 1992 to June 2015, with the training set from February 1992 to March 2015 and the test set from April 2015 to June 2015, as shown in Table 10. This dataset was selected due to its comprehensive temporal coverage and the complex patterns inherent in industrial production data. Such characteristics challenge the model’s ability to capture and predict long-term dependencies and fluctuations in data, an ideal test for GEP-SVM’s predictive robustness and long-term prediction.

The other dataset is from the literature [28], focusing on the short-term prediction of urban traffic flow, with data collected every 15 min from 0:00 to 24:00. Two months of data were selected as the training set, and 7 days of data were selected as the test set, as shown in Table 10 and Table 11. This dataset tests the GEP-SVM’s ability to adapt and perform in scenarios requiring rapid model adjustments based on real-time data, thereby assessing its efficacy in short-term predictive scenarios.

The experiments in this section focused on the performance differences of the GEP-SVM algorithm model in short-term and long-term prediction.

### 4.3. Algorithm Analysis

Future time shipments and a section of traffic flow were used as prediction counterparts, respectively, with the KNN, SVM, GEP, and GEP-SVM algorithms used to establish long-term and short-term prediction models. The four groups of models were analyzed, as shown in Table 12 and Table 13. As can be seen in Table 12 and Table 13, the GEP-SVM prediction model outperforms other single-prediction models based on the analysis of the performance metrics MSE and MAPE. In long-term prediction, the superiority of the GEP-SVM model is more pronounced than in short-term prediction. In short-term prediction, the prediction error values of GEP-SVM and GEP are similar. The experiment demonstrates that GEP-SVM can effectively improve prediction accuracy and precision, indicating its effectiveness and practicality.

## 5. Discussion

In the realm of agricultural pest management, the integration of machine learning and IoT technologies has emerged as a promising approach for predicting pest occurrences, thereby enhancing decision-making processes for farmers and reducing the reliance on frequent field inspections and excessive pesticide usage. Recent studies in this domain include the following works.

Marković et al. (2021) [29] and Saleem et al. (2021) [30] established the foundational groundwork by employing machine learning algorithms and IoT-based systems, respectively, achieving commendable accuracies in predicting pest appearances. However, these studies were primarily focused on binary outcomes (the presence or absence of pests) and were constrained by the short prediction horizons of five days and the specific focus on whitefly attacks on cotton crops. Building on this foundation, Tsai et al. (2023) [31] introduced a method that leverages transfer learning technology for time series feature extraction, which broadened the scope to multiple types of crop pests. This approach marked a significant advancement in capturing the dynamic nature of pest populations, but the study still grappled with the challenge of achieving high accuracy across diverse pest species. Saleem et al. (2023) [32] further expanded the predictive capabilities by utilizing a DNN model within an IoT framework to achieve a weekly pest prediction with an impressive accuracy of 94%. However, the generalizability of the model to different crops and pest types remains an area for further exploration.

Our research introduces the GEP-SVM model, which establishes a novel approach for predicting the occurrence of wheat blossom midges. This model not only contributes innovative ideas and valuable benchmarks to the field but also demonstrates a significant improvement in prediction accuracy. Specifically, the GEP-SVM model achieves a back-generation accuracy of 90.83%, which notably exceeds the performance of traditional models, such as K-nearest neighbor, naive Bayes, and back-propagation neural networks. This superior accuracy is derived from the model’s enhanced fitting and generalization capabilities, which are crucial for adapting to the complex dynamics of agricultural pests.

Importantly, the adaptable nature of the GEP-SVM model suggests its potential applicability beyond its initial implementation. The model’s robust generalization capabilities make it a promising tool for application in different agricultural settings, including various regions and crops afflicted by different pest species. For instance, the model could be tailored to predict the occurrence of pests in crops like rice or corn by adjusting the input parameters to reflect the specific ecological and climatic conditions of these new contexts. Additionally, the model’s flexibility and high accuracy offer a significant advantage for its deployment in diverse geographical regions where pest behavior may vary substantially, thus supporting a broader range of agricultural pest management strategies.

The contributions of our research extend beyond the specific context of wheat blossom midges, as they enhance the growing body of literature advocating the integration of genetic algorithms and machine learning in solving complex agricultural problems. Our work aligns with studies such as those by Al-Anni (2017) [33] and Aquino et al. (2017) [34], which have documented the benefits of employing genetic programming and machine learning techniques in prediction. Employing genetic programming and machine learning in prediction offers several benefits, such as improved accuracy and efficiency in handling complex data. Genetic programming, as demonstrated in Aquino et al.’s study, and machine learning, particularly genetic programming, as shown by Al-Anni et al., can effectively predict cancer recurrence by analyzing gene expressions from microarray data, thus providing reliable prognostic tools.

Overall, this study not only offers a practical solution to a pressing agricultural challenge but also contributes to the scientific discourse on the application of machine learning in agriculture, pushing the boundaries of current predictive modeling techniques. Furthermore, our study makes a methodological contribution by combining the strengths of GEP and SVM to create a robust tool for real-time and accurate pest prediction. This innovative approach not only improves the predictive accuracy but also provides a more nuanced understanding of the underlying patterns and factors influencing pest dynamics.

## 6. Conclusions

In this study, we developed a novel approach by integrating GEP with SVM to predict the occurrence of wheat blossom midges. The GEP-SVM model marks a significant enhancement in predictive accuracy within smart agriculture and pest prediction methodologies. Key conclusions from this study include the following:

1. Enhanced Prediction Accuracy: The GEP-SVM model significantly outperforms existing common models in accuracy, establishing a new benchmark for pest prediction in wheat.

2. Scalability and Adaptability: Preliminary tests indicate that the model can be effectively adapted to different datasets and pest types, suggesting its potential for broader application.

In summary, this study addresses the identified gap in the literature by proposing the GEP-SVM model, a novel and more accurate model for pest prediction, and establishes a new benchmark for future research in this domain. The success of the GEP-SVM model opens avenues for further exploration and the further application of hybrid computational models in agriculture and beyond, promising significant implications for sustainable farming practices and food security.

As we continue to refine and validate our model across different datasets and pest types, we anticipate its integration into practical agricultural decision-making processes, thereby enhancing the efficiency and effectiveness of global pest management strategies. Furthermore, for future work, we suggest exploring the integration of additional data sources, such as soil properties, plant phenological data, and genetic information, to enhance the model’s predictive accuracy. Additionally, testing the model on different crops and regions will be crucial to evaluate its generalizability and adaptability to diverse agricultural contexts. These efforts will contribute to the development of more robust and universally applicable pest prediction models, further advancing the field of smart agriculture and pest management.

## Figures and Tables

**Table 1:** Definitions and descriptions of variables used in the fitness function formula.

$T_{j}$	Target value on fitness samples
$C_{\left(i,j\right)}$	Chromosome return value on the fitness sample
M	Selection range

**Table 2:** Overview of three transfer operators and their functional characteristics in genetic algorithms.

Transfer Operator	Transfer Factor	Target Location
Gene transfer	Entire gene	Multigene chromosome
Insertion sequence elements (IS elements)	Short sequences where the first position is a function or endpoint	Gene head anywhere except the root
Root insertion sequence element (RIS element)	Short sequence where the first position is a function	Root of the gene

**Table 3:** Algorithm 1 GEP-SVM pseudo-code.

Input:		
	*cases*	The sample data set.
	*N*	Population size.
	*h*	Gene head length.
	*e*	Gene tail length.
	*n*	Maximum number of operations of the function.
	*k*	The number of genes.
	*MaxGeneration*	Fitness of termination iteration.
	*P_mu_*	The mutation probability
	*P_tr_*	Probability of string insertion
	*P_re_*	Recombination probability
	*P_ex_*	Extraction probability
Output:		
	*Y*	Optimal individual (classifier)
1:	*Pretreat cases*;	
2:	*S = InitialPopulation*;	
3:	*Best_Ind = null*;	
4:	*m = MaxGeneration;*	
5:	*repeat*	
6:	*analyze chromosome*;	
7:	*fitness ()*;	
8:	*S = Selection(S)*;	
9:	*S = Mutation(S) by P_mu_*;	
10:	*S = Transpostion(S) by P_tr_*;	
11:	*S = Recombinations(S) by P_re_*;	
12:	*S = Extraction(S) by P_ex_*;	
13:	*S = Invertion(S) by P_in_*;	
14:	*S = Adjustment(S) by P_ad_*;	
15:	*Retain (Best_Ind)*;	
16:	*m = m − 1*;	
17:	*until m = 0*;	
18:	*return (Best_Ind)*;	

**Table 4:** Evaluation index of occurrence degree prediction model of wheat blossom midges.

Indicator	Expression
MSE	$MSE=\frac{1}{n}\sum_{i=1}^{i}{\left(y_{i}-\hat{y_{i}}\right)}^{2}$
MAPE	$MAPE=\left(\frac{1}{n}\sum_{i=1}^{i}\left|\frac{y_{i}-{\hat{y}}_{i}}{y_{i}}\right|\right)\times 100\%$

where $y_{i}$ is the true value and $\hat{y_{i}}$ is the predicted value. n is the number of predicted samples.

**Table 5:** Historical climate data and wheat midge occurrence in central Shaanxi from 1933 to 2010.

Year	Average Temperature						Average Rainfall						Annual Total Accumulated Temperature	Annual Total Precipitation	Incidence Level or Occurrence Level
	January	February	March	July	August	September	January	February	March	July	August	September			
1933	2.4	3.7	7.3	28.3	27.9	20.5	1.8	0.8	34.5	47.4	77.0	48.3	5409.4	285.2	1
1934	1.7	1.8	7.7	28.4	27.0	21.3	3.5	20.6	24.7	98.0	75.1	49.1	5312.2	527.7	1
…	…	…	…	…	…	…	…	…	…	…	…	…	…	…	…
2009	2.5	6.1	9.7	27.0	24.9	22.5	5.4	0.0	14.8	155.6	124.0	31.5	5522.6	482.5	2
2010	0.5	3.4	12.0	26.6	26.4	22.3	9.2	4.9	9.8	90.6	97.4	49.3	5534.0	593.0	1

**Table 6:** Algorithm 2 pseudo-code for pest and disease GEP-SVM occurrence prediction.

Input: training dataset T	
Output: prediction formula f	
1:	begin
2:	While there are still data in the data set T;
3:	Read w data from T;
4:	Add the first w-1 data to the GEP parameter table and add the remaining data to the target table;
5:	End while
6:	Initialize the GEP run; while
7:	End while Initialize the GEP run; while Terminate condition is met;
8:	Output the optimal chromosome in the population;
9:	end while
10:	Return the formula f found by the GEP.
11:	end

**Table 7:** Configuration parameters for the GEP-SVM hybrid algorithm.

Operating Parameters	Detailed Description
Evolutionary generation	1000
Population size	30
Fitness function	Mean Squared Error
Set of functions	+, −, ×, /, Sqrt, Exp, Ln, Abs, Sin, Cos
Organization of chromosomes	The gene head is 6 genes in length and the chromosome is made up of 5 genes
Mutation probability	0.044
Inversion probability	0.1
IS transformation probability, RIS transformation probability	0.1, 0.1
Single-point recombination probability, two-point recombination probability	0.3, 0.3
Recombination probability, gene change probability	0.1, 0.1
Connection function	+

**Table 8:** Comparative analysis of prediction errors for wheat aphid infestation across different models.

Model	Training Set		Test Set	
	MSE	MAPE	MSE	MAPE
GEP-SVM	**1.57**	**5.51%**	**4.39**	**16.57%**
GEP	1.89	5.98%	4.75	16.93%
SVM	5.33	10.53%	6.78	17.49%
K Nearest Neighbors	5.69	11.16%	6.83	17.83%
Simple Bayes	5.51	10.91%	6.46	17.62%
BP Neural Network	5.74	11.77%	8.58	18.93%

**Table 9:** Performance metrics for models predicting wheat aphid occurrence in training and test datasets.

Model	Train/Test	M	D	AC	Precision	Recall	F1-Score	MCC
GEP-SVM	**Train**	**49**	11	**90.83%**	**0.870**	0.889	**0.880**	**0.501**
GEP		48	10	88.33%	0.829	**0.906**	0.866	0.477
SVM		47	11	87.50%	0.826	0.883	0.853	0.440
K Nearest Neighbors		46	12	86.66%	0.804	0.880	0.840	0.412
Simple Bayes		45	12	85.00%	0.782	0.878	0.827	0.386
BP Neural Network		46	12	86.66%	0.787	0.902	0.840	0.424
GEP-SVM	**Test**	**15**	2	**88.89%**	**0.857**	**0.923**	**0.888**	**0.563**
GEP		13	3	80.55%	0.714	0.909	0.800	0.395
SVM		13	3	80.55%	0.769	0.833	0.800	0.350
K Nearest Neighbors		12	4	77.78%	0.642	0.900	0.750	0.328
Simple Bayes		12	4	77.78%	0.692	0.818	0.750	0.268
BP Neural Network		11	5	75.00%	0.615	0.800	0.695	0.194

Note: M is the number of samples in which the actual and predicted values of wheat aphid incidence are equal, D denotes the number of samples in which the actual and predicted values differ by one level, and AC is the accuracy rate.

**Table 10:** Historical data on manufacturing shipments in the United States (1992–2015).

Time	Actual Value of Shipments (in Millions of USD)
1992.2	11,567
1992.3	11,345
1992.4	11,987
1992.5	11,674
…	…
2015.4	482,323
2015.5	481,347
2015.6	484,363

**Table 11:** Time-specific traffic flow data for urban trolley systems.

Time	Traffic Flow (in Trolleys)
7:00–7:15	141
7:15–7:30	138
7:30–7:45	147
7:45–8:00	155
8:00–8:15	167
8:15–8:30	233
8:30–8:45	245
8:45–9:00	288
…	…
19:15–19:30	267
19:30–19:45	221
19:45–20:00	216

**Table 12:** Comparative analysis of prediction model performance on manufacturing shipments’ data.

Model	MSE	MAPE
KNN	1187	0.0037
SVM	688.4	0.0026
GEP	564.2	0.0017
GEP-SVM	90.3	0.00098

**Table 13:** Evaluation of Prediction Models on Traffic Flow Data Using Error Metrics.

Model	MSE	MAPE
KNN	1.86	0.0369
SVM	1.47	0.0253
GEP	1.32	0.0350
GEP-SVM	1.29	0.0290

## Data Availability

The raw data and source codes supporting the conclusions of this article will be made available by the authors on request.
